# TEMPORAL DYNAMICS OF UNMET LONG-TERM CARE NEEDS IN CHINA: AN AGE PERIOD COHORT ANALYSIS

**DOI:** 10.1093/geroni/igae098.0441

**Published:** 2024-12-31

**Authors:** Jingwen Zhang

**Affiliations:** University of Sheffield, Sheffield, England, United Kingdom

## Abstract

The rapid demographic changes, combined with heavy reliance on informal care, pose significant challenges to meeting long-term care (LTC) needs in China. However, research has yet to comprehensively appraise how and to what extent the unmet need has changed during the past two decades. Drawing on data from 6,030 urban and 5,070 rural residents in the Chinese Longitudinal Health Longevity Survey (CLHLS), 2005-2017/18, this study investigates the temporal changes in unmet LTC needs among Chinese older adults. We applied the newly developed age-period-cohort interaction (APC-I) model to disentangle the three temporal processes and their place-based rural-urban variation. The study found that, overall, rural older adults experienced a higher risk of unmet needs for LTC, yet the age, period and cohort effects on unmet needs among rural older people differed from their urban counterparts. Although ‘younger’ older adults had fewer care needs than older adults, they had a higher risk of experiencing unmet needs. The variation in the age effects was larger among rural older adults. The risk of having unmet needs did not change significantly over the 12 years. The unmet needs for LTC were more pronounced among more recent cohorts than previous generations, especially in urban areas. The findings provide crucial policy insights, underscoring the necessity for targeted interventions to address the care needs of ‘younger’ older adults, whose demands may not be perceived as severe enough for immediate attention. The escalating care gap among recent cohorts emphasises the need for increased investment in the formal LTC system.

